# Development of Framework for Estimating Fatality-Related Losses in the Korean Construction Industry

**DOI:** 10.3390/ijerph18168787

**Published:** 2021-08-20

**Authors:** Jaehyun Lee, Jaewook Jeong, Jayho Soh, Jaemin Jeong

**Affiliations:** Department of Safety Engineering, Seoul National University of Science and Technology, 232 Gongneung-ro, Nowon-gu, Seoul 01811, Korea; archi0528@seoultech.ac.kr (J.L.); jhsoh95@seoultech.ac.kr (J.S.); ss96011@seoultech.ac.kr (J.J.)

**Keywords:** fatality loss, framework, productivity loss cost, compensation cost, business and administrative loss cost

## Abstract

The number of fatalities in the construction industry is highest among all industries; thus, various losses in the form of fatalities in construction represent a significant factor for safety management. If a fatality occurs, it is important to estimate the overall loss, as a significant financial loss occurs for each project participant. However, in studies of the cost of accidents involving a fatality conducted abroad, the framework cannot be applied as it is because insurance types, systems, and legal cost systems differ by country. Therefore, we developed a framework for calculating the fatality loss considering various aspects of labor, industry, and regulations in Korea. This was done in four steps: (i) data collection and criteria selection; (ii) proposed framework development; (iii) conduct of questionnaire surveys for the case study; and (iv) analysis and evaluation of the case study. As a result of the data analysis after the case study of general construction companies, the total fatality loss was estimated to be 2,198,260 USD/person. The contributions of this study are the development of a framework composed of newly discovered items that can acquire reliable data in consideration of the properties of the construction industry.

## 1. Introduction

The construction industry is considered to be very important to the economies of most developed and developing countries, as construction is one of the key contributors to national economic growth [1,2,3,4]. For example, according to the Bureau of Economic Analysis (2020), approximately 4.5% (USD 6760 billion) of gross production (USD 151,268) in the United States occurs in the construction industry as of 2019 [5]. Additionally, Shohet et al. [6] reported that the construction industry currently employs 6–10% of the world’s workforce.

However, in 2019, the International Labor Organization (ILO) reported that there were approximately 2.8 million industrial accidents and incidences of work-related diseases worldwide per year [7]. In addition, it has been found that the safety performance of the construction industry is low [8] and that the accident rate is higher than those in other industries [9,10]. According to the Bureau of Labor Statistics (2020), among all other high-risk sectors in 2019, the number of fatalities in the construction industry was highest at 1061 (19.9% of the total number) [5]. High-risk sectors include agriculture, manufacturing, transportation, and warehousing, and related administrative and support facilities.

According to Sunindijo and Zou [11], fatalities in the construction industry account for about 30–40% among all industries. This stems from the characteristics of what is considered a dangerous industry; specifically, the construction industry has relatively low safety performance when considering injury rates [12,13,14]. Lee et al. [15] reported that the number of fatalities continues to increase, with construction sites now considered among the most dangerous workplaces. In addition, Ho [16] reported that the construction industry exhibited higher fatality and injury rates compared to other industries due to its unique characteristics. He described its unique characteristics as including the factors of unpredictable field conditions, various human behaviors, and unsafe working procedures.

Within the global situation, the construction industry in Korea has grown rapidly over the past several decades [17,18]. In relation to this, efforts have been made to construct infrastructure and residential facilities to accommodate the rapid expansion of major cities [19]. Therefore, Korea is also experiencing the highest levels of accidents in the construction industry, which is considered one of the most dangerous occupations due to the dynamic and temporary nature of these workplaces. For example, construction workers are characterized as those who constantly change their work locations depending on the project and project stage. In addition, the complex working conditions of construction sites and the range of characteristics of the various types of ongoing construction work produce results that are not standardized, unlike the factories that characterize the manufacturing and nuclear industries [20,21].

In particular, Choi et al. [17] reported that Korea’s fatality rate is higher than those in other countries, with that number being nearly ten times higher than that of the United Kingdom (UK). Based on a report published by Choi et al. [17], our research team reviewed the fatality rate, defining it as the number of fatalities per 100,000 full-time equivalent workers by accident (as of 2017) in 35 countries for which information can be obtained among Organization for Economic Cooperation and Development (OECD) member countries. As a result, the average fatality rate for all industries in 35 countries was 1.87; for the construction industry, the rate was 6.03 (3.22 times that for industries overall). On the other hand, the average for all industries in Korea was 3.61, with the construction industry having a rate of 25.45 (7.05 times the total industry). Therefore, the average for the entire Korean industry was 1.93 times higher than that of the OECD, and the average rate in the construction industry was 4.22 times higher [22]. As such, it was judged that certain characteristics exist that make the impact of accidents involving a fatality was greater than those involving injuries, especially in Korea [22]. In addition, most countries in the world as well as Korea use the index of the number of fatalities when evaluating the safety performance of a company or country [17]. Therefore, the review conducted here focused on fatalities excluding injuries [22].

Within this backdrop, the Korean government has instigated a campaign entitled “Reduce industrial fatal accidents by half until 2022” as a national initiative. Nevertheless, according to the Korea Occupational Safety and Health Agency (KOSHA), the number of fatalities among construction workers has levelled off since 2000. In 2018, the construction industry experienced the most fatalities, accounting for 49.95% of all industrial fatalities.

In this situation, it is very important to calculate fatality losses by considering various aspects of the construction industry because if an accident involving a fatality occurs, it causes serious confusion with regard to the balance of the work environment [23]. In the event of a fatality at work, a significant financial burden is placed on national health agencies, employers (people who have to fight productivity declines, reduced profits and investment opportunities), and on the affected employees (loss of wages and poor quality of life, etc.).

Research on accident costs was pioneered by Heinrich [24]. He categorized costs into direct and indirect costs and emphasized the importance of indirect costs [25]. In addition to these traditional classification methods, several researchers have suggested different types of accident costs according to the characteristics of the expense. Riel and Imbeau [26] classified health and safety costs into the three categories of insurance-related costs, work-related costs, and perturbation-related costs. Additionally, from this perspective, such expenses can be classified into quantifiable costs, irreducible costs, and intangible costs. Rikhardsson and Impgaard [27] categorized accident costs into time, materials and components, external services, and other costs to simplify the process for management.

However, because the contents of these various studies have typically focused on methodologies used abroad, there are many limitations when applied to Korea. This is true because the characteristics of the Korean construction industry are not considered. For example, some previous studies have quantitatively calculated the fatality loss by considering the cost of insurance [12,28]. However, there are certain characteristics unique to the construction industry in Korea compared to those in other countries [29,30]. The construction industry in Korea has a type of social insurance classified as a form of health insurance, a national pension service, and employment insurance [29]. Moreover, there are other types of insurance such as an industrial accident insurance and workers’ compensation insurance [30]. Specifically, industrial accident insurance includes medical care benefits, the temporary disability compensation benefits, survivor benefits, and a funeral service expense benefit in Korea. On the other hand, most domestic-based studies did not comprehensively consider the characteristics of the construction industry, and there is no case in which the delayed reimbursement cost, which is a large loss in the case of a fatality, was considered. In this study, insurance types, systems, and legal cost systems that are differentiated from other countries were defined as Korean construction characteristics. Given these characteristics of the construction industry in Korea, a framework to calculate the fatality loss that also considers them is necessary.

On the other hand, Gavious et al. [31] emphasized the need to acquire robust and quantifiable information that can conclusively demonstrate the actual cost in order to develop a reliable way to assess the cost of a workplace accident. Rikhardsson and Impgaard [27] argued that a simple methodological basis that could be used by business owners in a short period of time was needed. In addition, Brody et al. [32] states that it is important to find a simple way to assess reliable indirect costs to ensure the quality and accuracy of observations without discouraging the company.

Therefore, this study attempts to develop a useful framework to calculate the fatality loss while also taking into account the characteristics of the Korean construction industry, focusing on construction projects and using reliable information. To do this, the research was conducted in the following steps:

(i) Data collection and criteria selection;
(ii) Proposed framework development;
(iii) Conduct of questionnaire surveys for the case study; and
(iv) Analysis and evaluation of the case study.

In particular, items that are differentiated from previous studies in the proposed framework development stage are summarized below:

- Framework optimization by applying the characteristics of the construction industry;
- Selecting reliable items that can be obtained from construction companies;
- Discovering and applying new items related to loss costs (delay reimbursement cost, etc.); and
- Developing a framework for calculating comprehensive loss costs.

## 2. Literature Review

As shown in Table 1, a summary of the variety of studies conducted thus far in an effort to quantify accident losses caused by industrial accidents is as follows: (i) studies that derive and calculate the accident loss for all industries including the construction industry; and (ii) studies that derive the accident loss in the construction industry and calculate the value.

First, for all industries including the construction industry, researchers have concentrated on deriving and calculating the fatality loss [23,27,33,34,35,36,37,38].

Lebeau et al. [33] quantified the costs of injuries and diseases in 26 industries in Quebec, Canada, from the perspective of the employer, worker, and community, taking into account the medical costs, funeral costs, and human costs. Among the employers, workers, and communities, workers were most responsible for the costs of injuries and diseases caused by industrial accidents. The construction industry was 15th out of 26 industrial groups incurring injury and disease costs due to industrial accidents. It was suggested that the results of the study could be helpful for those deciding the direction of research to prevent industrial accidents [33]. Leigh et al. [34] quantified the cost of injuries and illnesses in the United States in consideration of items such as medical care, lost productivity, and pain and suffering, and presented a ranking by industry. As a result of that study, the taxicab industry was the industry with the highest costs of injury and illness per worker. The authors suggested that their research results could serve as the basis for policy decisions by industry group [34]. Rikhardsson and Impgaard [27] developed a model for evaluating the costs borne by companies among occupational accident costs using a systematic accident cost analysis (SACA). Their developed model indicated that two-thirds of accident costs by the company were tangible expenses, with the other third being invisible expenses. The developed model can visualize the costs incurred by the company’s OHS department [27].

Additionally, Shalini [37] measured the accident cost of work in Mauritius, a small island country, considering the loss of productivity, medical costs, and safety investment costs. Through that study, it was confirmed that the cost of work-related accidents in a small developing country is higher than that in other countries [37]. Xiang et al. [38] conducted an analysis to determine whether there was a difference in injury costs due to industrial accidents between American workers and immigrant workers using data on compensation costs for industrial accidents. The probability of receiving treatment for immigrant workers in industrial accidents was 75.6%, and the probability of U.S. workers receiving treatment was 77.3%. In addition, it was confirmed that the average medical cost per injured worker was USD 2357 for U.S. workers and USD 2351 for immigrant workers [38].

The above-mentioned studies derived accident loss items for all industrial groups including the construction industry and quantified their values. However, unlike other industrial groups, in the event of an industrial accident, the construction industry incurs various additional costs such as short-term recovery costs, institutional penalties and fines, making it necessary to calculate the accident loss in consideration of the characteristics of the construction industry.

Second, for the construction industry, researchers have concentrated on deriving and calculating the fatality loss [39,40,41,42,43,44,45,46,47,48,49,50]. Allison et al. [39] quantified the cost of accidents in the Australian construction industry from the perspectives of employers, workers, and the government, taking into account the production disturbance costs, human capital costs, and medical costs. Their study found that employers paid the highest accident cost with a short absence, while the government paid the highest accident cost with a long absence [39]. Feng et al. [41] investigated the accident costs in the construction industry and determined how much it affected the metric known as the contract sum. As a result of their study, the average direct accident cost, indirect accident cost, and total accident cost accounted for 0.165%, 0.086%, and 0.25% of the contract sum, respectively. In addition, it was confirmed that the ratio of the average indirect accident cost and the average direct accident cost was 1:1.92 through the research results [41].

Gholizadeh and Esmaeili [43] quantified the accident cost of electrical work and verified through ANOVA that there was a difference in the accident cost according to the accident type, building type, and construction cost. As a result of that study, it was verified that there is a statistical difference between the accident cost according to the accident type and the accident cost according to the building and the building type. However, the difference in accident cost according to the construction cost was not verified [43]. Schoonover et al. [47] quantified the prevention index (PI) by type of construction and the severity of accidents through the cost of accident compensation claims of U.S. construction workers. In that study, it was confirmed that the ‘Foundation, structure, and building exterior contractor’ among all construction factors represented the highest cost in terms of both the PI and accident severity [47]. Waehrer et al. [50] presented fatality and injury costs by considering the direct and indirect costs that were not limited to workers’ compensation costs. As a result of their study, the cost per case of deaths and injuries in the construction industry was found to be USD 27,000, twice the average cost of industry overall [50].

As described above, previous studies have quantified the fractional cost item, not the overall cost item, for accident losses in the construction industry. In addition, it was judged that the reliability of the supporting data was insufficient for the quantified cost items overall. In addition, because previous studies defined cost items taking into consideration of the characteristic of each country, the cost items can be used for the construction industry in each respective country, but not outside it. However, although the specific characteristics of the Korean construction industry were not considered, it is believed that these limitations would arise when applying these items as they are.

## 3. Materials and Methods

**Figure 1:**
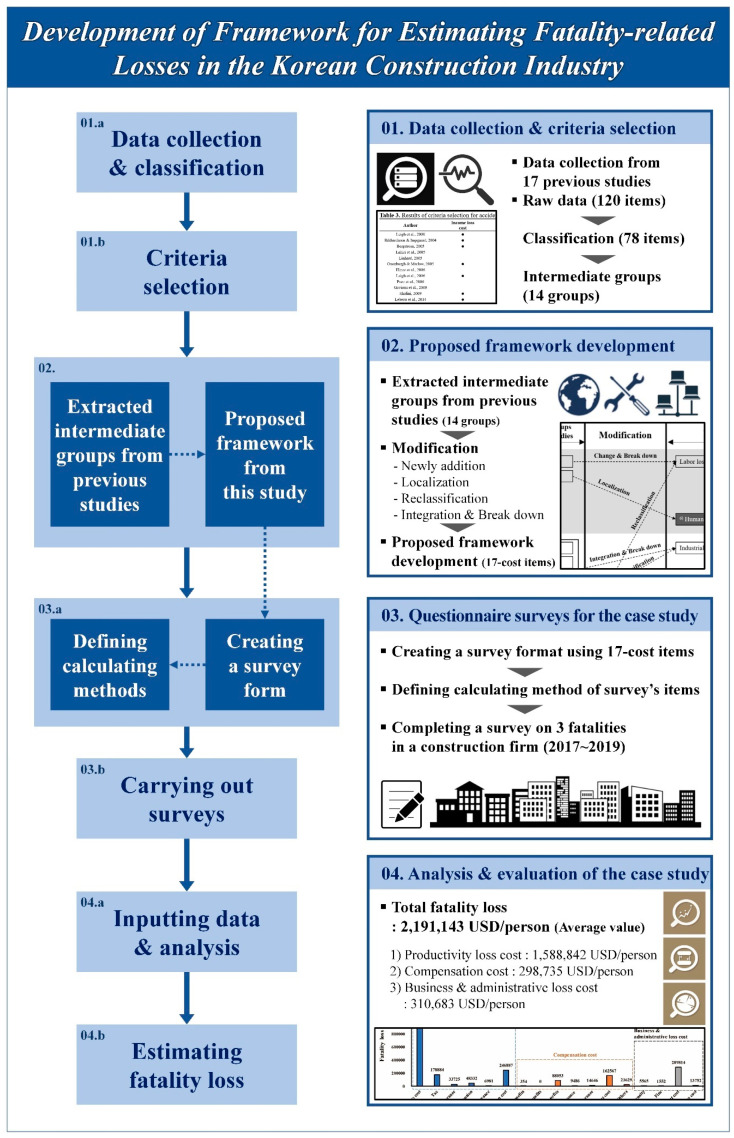
Research framework.

Figure 1 shows the framework of this study carried out via the aforementioned four-step process.

In the first step, data related to fatality losses are collected from the literature review and then classified by content. Criteria for calculating the actual fatality loss value are selected through these classified groups.

As a second step, the intermediate groups in the previous research were derived based on the selected criteria. In order to consider the characteristics of the construction industry and calculate the fatality loss with high reliability, the proposed framework was developed by modifying the cost items of the intermediate groups in the previous research. To do this, focus group interviews were conducted with four different construction company experts in Korea. To select the four experts, an official letter related to the focus group interviews was sent to the safety-related teams of the top ten construction companies in Korea, requesting their participation. Focus group interviews were conducted with the four who accepted the invitation.

In the third step, the survey information was created based on the cost items of the proposed framework in this study. When creating the survey form, a method to quantify the fatality loss was prepared by defining the numerical relationship between the input data and the output data for each cost item. In this case, as a case study, the survey was tailored to large construction companies.

As a fourth step, after coding the survey results, the input data are analyzed through the calculation methods defined in this study. As a result of the data analysis in the case study, the average total costs related to a fatality loss (USD/person) can be estimated.

### 3.1. Data Collection and Criteria Selection

As the first task in the selection of the criteria used to calculate the fatality loss, previous studies dealing with accident loss were analyzed. Seventeen previous studies over the last two decades were found to be relevant to accident losses. Overall, 120 accident loss items were found initially, and 78 cost cases were organized after integrating similar cases. Here, it was possible to reduce the number to 78 items by integrating items with similar contents among the 120 data instances. To this end, the items were integrated by synthesizing keywords, categories, and meanings, and all 120 data items could be encompassed by the 78 items without excluding any data.

We grouped items that could be collected into the same category again. For example, we grouped the following eight items into one group: lost earnings, normal working time, wage cost, long-term disability, injured worker productivity losses, lost income, salary cost, and sick leave. In addition, the six items of consultants and legal support, intervention costs, legal fees, legal expenses, the cost of judicial proceedings, and costs arising from possible labor disputes were placed in the same group.

Subsequently, these intermediate groups were utilized as baseline criteria to establish the proposed framework of fatality loss. The final cost items in this study are presented in detail in Section 4.1.

### 3.2. Proposed Framework Development

The process of deriving the proposed framework in this study is as follows. First, the intermediate groups can be divided into three relatively broad ranges: the productivity loss cost, compensation cost, and business and administrative loss. The productivity loss cost consists of the income loss cost and the human cost, and the compensation cost includes the medical cost, medical leave wages, survivor’s benefits, the funeral cost, the compensation cost, time and productivity loss, material loss, and financial loss. In addition, the business and administrative loss cost consists of the intervention cost, penalty, fine, and administrative loss cost.

However, these intermediate groups are insufficient to calculate the fatality loss reliably, which is the goal of this study. In this case, it is uniquely necessary to calculate the criteria considering the characteristics of the Korean construction industry. Second, different countries may have different compensation systems for different accidents. Thus, institutional penalties that can affect the fatality loss in the construction industry may vary depending on the country.

Therefore, the necessity of deriving a feasible framework to obtain reliable data considering the characteristics of the Korean construction industry was raised. To realize this, focus group interviews were conducted with Korean construction safety experts. When selecting the safety experts, we considered that they should have 15 years of experience on safety-related teams at different construction companies and that they should hold the position of manager or higher. Based on this, the intermediate groups pertaining to fatality loss were revised in the following way. The items of the proposed framework considering the above are summarized below.

- Added: Social insurance (health insurance, national insurance, employment insurance), workers’ compensation insurance, delay reimbursement costs;
- Change and break down: labor loss cost (income loss cost, tax, social insurance);
- Localization: human cost, administrative loss cost;
- Reclassification: labor loss cost, industrial accident insurance; and
- Integration and breakdown: industrial accident insurance (medical care benefits, temporary disability compensation benefits, survivor benefits, funeral service expenses), others (e.g., time loss, material loss), legal fees (penalties, fines).

In conclusion, the proposed framework for estimating the fatality loss value in construction was reorganized by 17-cost items.

### 3.3. Conduct of Questionnaire Surveys for the Case Study

A questionnaire survey for the case study was utilized to apply the proposed framework. Table 2 summarizes the required information for the survey based on the proposed framework. The hierarchy of the required information is mainly divided into three tiers.

The first tier contains information about the construction company, with seven questions. The second tier accounts for accident information, with nine questions, and the third tier has items about the cost of the loss resulting from the occurrence of a fatality. It consists of a total of ten questions and covers industrial accident insurance.

In order to increase the reliability of the survey, the final survey format was completed after several revisions were made through focus group interviews with several experts at a Korean construction company.

After completing the questionnaire survey format, the questionnaire survey was applied in the case studies with the proposed framework. We presented the questionnaire survey to the general contractor (hereafter the ‘company’) to obtain information on the cost items and fatalities. The companies selected were in the top ten groups based on sales in the Korean construction industry and thus offer representativeness with regard to the questionnaire survey target. The questionnaire survey response rate was calculated using the number of companies that answered the questionnaire survey. The questionnaire survey was conducted at one company, and that company responded to the questionnaire survey (response rate: 100%). A total of three fatal accidents occurred at the company over three years (2017 to 2019). The target company is considered to be representative because the fatality rate for the company among the top ten construction companies in Korea was most similar to that of the Korean construction industry regarding the number of fatalities per 100,000 full-time equivalent workers by accident.

### 3.4. Analysis and Evaluation of the Case Study

After collecting the results of the questionnaire, they were evaluated and the case study proceeded. Table 3 shows the calculation method for the 17 cost items pertaining to fatality loss. Each cost item was analyzed using the three aforementioned tiers. Some cost items were obtained through the survey directly. Others can be calculated using national statistical data. The other cost items were calculated by the proposed method using raw data from the survey. For the calculation method, nine cost items were obtained through the survey, one cost item was calculated using statistical data, and seven cost items were calculated using data from the survey.

First, cost items such as medical care benefits, temporary disability, compensation benefits, survivor’s benefits, funeral service expense, workers’ compensation insurance, settlement cost, others, penalty, and fine were analyzed according to the survey.

Second, the human cost was analyzed by calculations using national data. There is no previous research that quantifies the human cost incurred by accidents in the construction industry. In the Health and Safety Executive (HSE) case in the UK, the cost of the reduced quality of life of the deceased due to a traffic accident was used to calculate the human cost [51]. In South Korea, a study by the Korea Transport Institute (KOTI) quantified the cost of the reduced the quality of life of the deceased due to traffic accidents [52]. Therefore, the human cost in this study was applied by referring to the cost of the lowered quality of life as calculated by KOTI.

Third, cost items such as the income loss cost, tax, health insurance, national pension, employment insurance, the delay reimbursement cost, and the administrative loss cost were collected through the survey once more, after which they were calculated and analyzed. The income loss cost was calculated as the net present value, taking into account the wage increase rate and the real discount rate for the worker wage, which was expected to be received by the deceased worker until retirement (age 65) [33,37,39,45]. In Korea, the retirement age of workers is defined as the age of 60 or older. However, due to the development of medical technology, the ratio of workers who are older than age of 60 has been steadily increasing in Korea [53]. Additionally, various studies have defined the retirement age of workers as 65 [33,37,39,45]. Therefore, the retirement age was defined as 65 years of age. Tax was calculated as the net present value, taking into account worker income, tax rates, and progressive taxes as the amounts expected to be paid each year until the deceased worker retires [54]. Health insurance was calculated as the net present value, taking into account worker income and health insurance premium rates as the amounts expected to be paid annually until the deceased worker retires [55].

Furthermore, the national pension was calculated as the net present value, taking into account the worker income and the national pension premium rates as the amounts expected to be paid annually until the deceased worker retires [55]. Employment insurance was calculated as the net present value, taking into account worker income and employment insurance premium rates as the amounts expected to be paid annually until the deceased worker retires [55]. The delay reimbursement cost refers to the amount needed to compensate for the delay construction period due to an accident. This cost was calculated by taking into account the accident work construction cost of the stop-work period, the ratio of the labor cost, the wage premium and the manpower premium [56]. The administrative loss cost was calculated by considering the number of participants, the number of days, and worker wages as the amounts incurred to investigate accidents.

## 4. Results and Discussion

### 4.1. Results of Criteria Selection from Previous Studies

As a result of grouping 78 cost cases previously, as shown in Table 4, they could be classified into a total of 14 intermediate groups as follows: the income loss cost, human cost, medical cost, medical leave wage, survivor benefit, funeral cost, compensation cost, time and productivity loss, material loss, financial loss, intervention cost, penalty, fine, and administrative loss cost. These 14 intermediate groups were grouped based on the general cost management work breakdown structures of Korean construction projects. This was done because when developing survey items for a case study, this method can serve as a basis for increasing the reliability of the acquired data.

Table 5 shows the results of a comparative analysis between the proposed framework and those in previous studies focusing on fatality loss. The number of cost items covered in the previous studies can be ranked as follows: ten items for Feng; eight items for Linhard; and seven items for Leigh et al., Lebeau et al., and Ibarrondo-Dávila et al. [28,33,41,44,49].

The theme of cost items appearing in the literature review can be organized as follows: 16 studies on medical cost, 13 studies on time and productivity loss, nine studies on financial loss, eight studies on income loss cost, eight studies on administrative loss cost, seven studies on material loss, and six studies on intervention cost. The other theme associated with the aforementioned seven cost items was found to exist in four studies. The result of the focus group interviews indicated that all of the remaining seven cost items should be retained to calculate the fatality loss considering various aspects.

Therefore, 14 cost items covered in the previous studies were included to develop the framework proposed in this study. As a result, it was possible to evaluate and calculate the fatality loss comprehensively by utilizing more cost items as criteria than in previous studies [28,33,41,49].

### 4.2. Results of Proposed Framework Development

**Figure 2:**
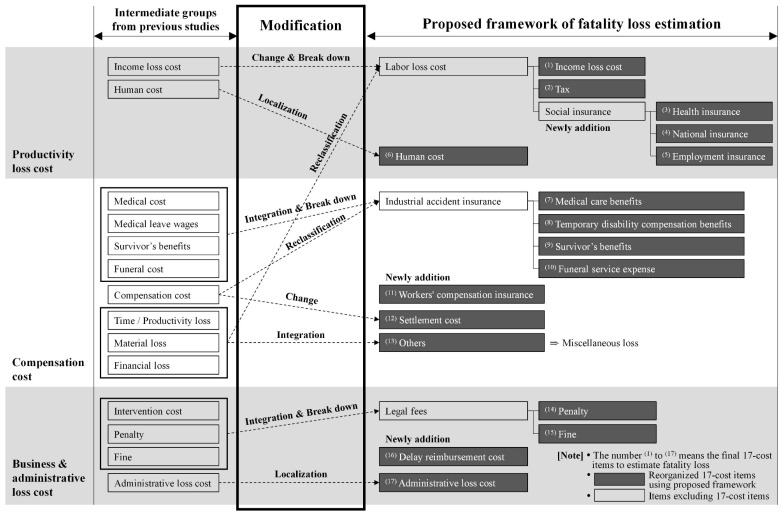
The proposed framework for fatality loss estimation.

Figure 2 shows the process of deriving the proposed framework in this study. As mentioned in Section 3.2, the intermediate groups for fatality loss were revised as follows: ‘Added’, ‘Change and Breakdown’, ‘Localization’, ‘Reclassification’, and ‘Integration and Breakdown’.

The proposed framework with greater reliability considering the characteristics of the construction industry was composed of a total of 17 cost items, as mentioned in Section 3.2. An explanation of each cost item is given in Table 6.

The 17 cost items consisted of the following: ^(1)^ income loss cost, ^(2)^ tax, ^(3)^ health insurance, ^(4)^ national insurance, ^(5)^ employment insurance, ^(6)^ human cost, ^(7)^ medical care benefits, ^(8)^ temporary disability compensation benefits, ^(9)^ survivor’s benefits, ^(10)^ funeral service expense, ^(11)^ workers’ compensation insurance, ^(12)^ settlement cost, ^(13)^ others, ^(14)^ penalty, ^(15)^ fine, ^(16)^ delay reimbursement cost, and ^(17)^ administrative loss cost.

The productivity loss cost is composed of the sum of the labor loss cost and the human cost. The labor loss cost is classified into the income loss cost, tax, and social insurance costs. Again, social insurance is classified into the health insurance, national pension, and employment insurance types. In particular, tax and social insurance are items used to calculate the loss cost from the government side and are considered to represent the differentiation of this paper because they are cost items that consider the characteristics of Korea, which have rarely been covered in previous papers.

Health insurance is a social security system that allows workers to receive medical services in order to prevent excessive burdens on households due to high medical expenses caused by an illness or injury. Citizens usually pay insurance premiums, and the National Health Insurance Corporation (NHIC), the insurer, manages them and operates the service, providing insurance benefits when necessary, so that citizens can share risks and receive necessary medical services.

The national pension is a public pension system operated directly by the government. It is operated based on insurance premiums paid when individual citizens engage in income activities. It is a pension system that provides some maintenance to people by paying pensions to the person or their family when income activities are stopped due to age, death, or a disability caused by a sudden accident or illness.

Employment insurance is a social insurance policy to ensure life stability in case workers lose their jobs. In addition to the unemployment benefit program that pays wages for a certain period of time, the employment security program and the vocational competency development program are implemented to promote re-employment and prevent unemployment through the development and improvement of vocational skills for job seekers and by providing active job placement services.

Human cost is defined as the cost of lowering the quality of life of workers occurred by fatal and injury accident. It can be divided into the cost incurred by workers and cost incurred by the workers’ family. However, because this study considered only fatal accidents, human cost in this study was defined as the costs of mental pain, sadness, or pressure of the bereaved family members of a worker who died in an accident.

The compensation cost consists of the sum of industrial accident insurance, workers’ compensation insurance, settlement costs, and others. Industrial accident insurance here consists of detailed categories of medical care benefits, temporary disability compensation benefits, survivor benefits, and funeral service expense. In particular, industrial accident insurance and workers’ compensation insurance are among the items that are differentiated from previous papers. These costs reflect the characteristics of Korea’s insurance system and account for a large proportion of the compensation costs for injured workers. Compensation costs are linked to losses by the state or employer.

Industrial accident insurance is a mandatory form of insurance that the state is responsible for to ensure the livelihoods of workers involved in an industrial accident and their families. In this system, the state collects a predetermined insurance premium from the employer and compensates industrially injured workers on behalf of the employer with these financial resources.

On the other hand, workers’ compensation insurance is a type of liability insurance instead of a mandatory type of insurance. It is insurance that compensates for damages under the legal liability that the employee must bear additionally in case a worker employed at a certain workplace suffers a sudden accident during work.

The business and administrative loss cost is defined as the sum of the business loss cost and the administrative loss cost. The business loss cost consists of the sum of legal fees and the delay reimbursement cost. Again, legal fees are divided into penalties and fines. In the event of a fatality during a project in the construction industry, construction can be halted for several weeks. In such cases, the construction period will be delayed as long as the construction interruption period. To make up for this, the employer has to rush the construction work. For rushed construction work, as night work or holiday work is generally performed, labor costs and product premiums are incurred compared to the original labor costs. These costs generally lead to losses for the employer. This cost varies depending on the properties and conditions of the project but accounts for a significant proportion of the business and administrative loss cost.

However, previous papers did not consider the delay reimbursement cost as a loss related to a fatality [27,57,58]. Therefore, the delay reimbursement cost is part of the cost of the fatality loss and thus represents the characteristics of the Korean construction industry. It is considered as a differentiated item in this article.

### 4.3. Results of the Estimated Fatality Loss by Case Study

**Figure 3:**
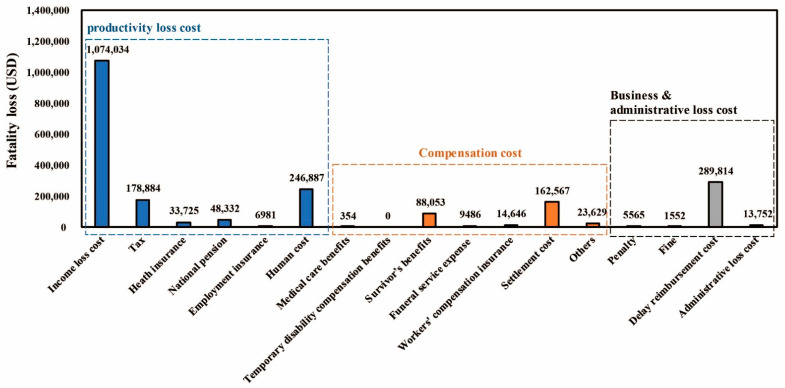
Total average of the fatality loss for 17 cost items.

A case study of three fatalities in construction that occurred at three sites in Korea was conducted with the proposed framework. The results were analyzed by acquiring evidence and raw data for each accident. Figure 3 and Table 7 show the results of the case study using the proposed framework.

The subtotal of the productivity loss cost was estimated to be 1,588,842 USD/person. The average values of the income loss cost, tax, health insurance, national insurance, employment insurance, and human cost were calculated as 1,074,034 USD/person, 178,884 USD/person, 33,725 USD/person, 48,332 USD/person, 6981 USD/person, and 246,887 USD/person, respectively.

The subtotal of the compensation cost was estimated to be 298,735 USD/person. The average values of medical care benefits, survivor benefits, funeral service expense, workers’ compensation insurance, settlement cost, and others were calculated as 354 USD/person, 88,053 USD/person, 9486 USD/person, 14,646 USD/person, 162,567 USD/person, 23,629 USD/person, respectively.

The subtotal of the business and administrative loss cost was estimated to be 310,683 USD/person. The average values of penalties, fines, the delay reimbursement cost, and the administrative loss cost were calculated as 5565 USD/person, 1552 USD/person, 289,814 USD/person, and 13,752 USD/person, respectively.

In conclusion, as a result of estimating the total fatality loss for three fatalities in the construction industry, it was estimated to be 2,198,260 USD/person.

**Figure 4:**
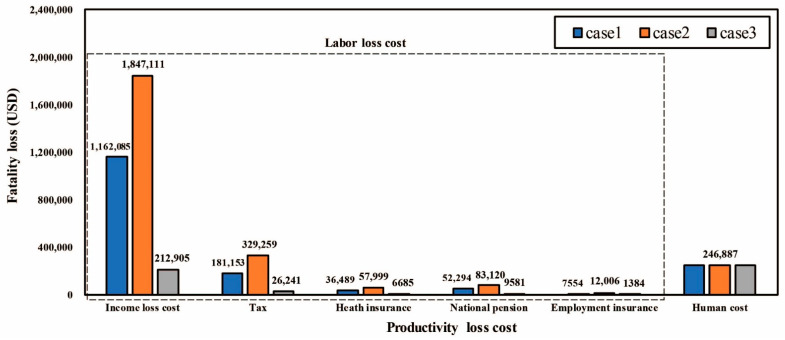
Comparison of productivity loss cost.

Figure 4 is a comparison of the productivity loss cost for the three fatalities above. The cost that accounts for the largest proportion is the income loss cost. Regarding the income loss cost, it was found that the deviation was large depending on the age of the deceased. As the age of the deceased is lower, the income loss cost increases. In other words, the two variables are inversely proportional to each other because the younger the person in such a case, the greater the cumulative value of the wages earned until retirement. In addition, tax, health insurance, national insurance, and employment insurance all have characteristics that are calculated as dependent variable values according to the independent variable of age, akin to the income loss cost.

**Figure 5:**
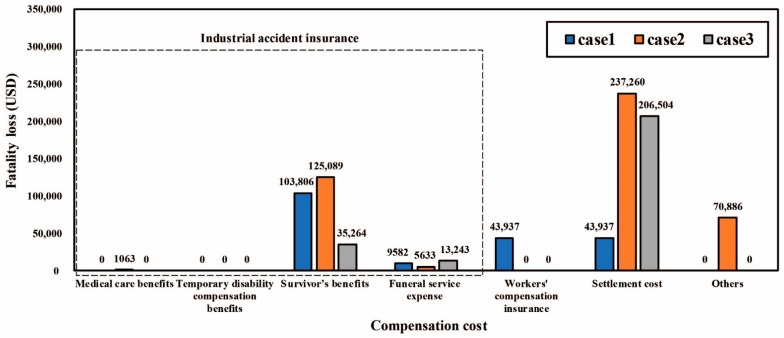
Comparison of compensation cost.

Figure 5 presents a comparison of the compensation cost for the three fatalities. Among them, the settlement cost and survivor benefits were the largest costs. For the settlement cost, because there is no legal standard, there was a large difference in value for each construction project. In addition, regarding survivor benefits, one characteristic is that the deviation increases according to the number of survivors.

**Figure 6:**
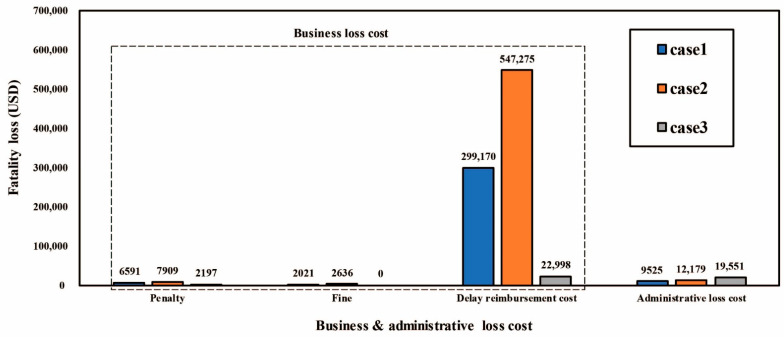
Comparison of business and administrative loss cost.

Figure 6 is a comparison of the business and administrative loss cost for the three fatalities. Among them, the delay reimbursement cost was found to be the largest. This cost is the most important loss incurred in the event of a fatality in the construction industry. The delay reimbursement cost increases when the construction cost of an accident occurring during employment is high, the range of interruptions is wide, and there are many working days. Therefore, one characteristic is the large variation depending on the conditions of the construction site.

## 5. Conclusions

The construction industry has the highest fatal accident rate compared to other industries. Additionally, when an accident involving a fatality occurs at a construction work site, a considerable financial burden is placed on the worker, employer, and government. There is, however, the lack of a feasible quantitative calculation method by which to determine the fatality loss imposed on the worker, employer, and the government while properly reflecting the characteristics of the construction industry in Korea. Therefore, it is important to calculate the fatality loss by considering various aspects of the construction industry. Thus, this study proposed a framework to calculate the fatality loss while considering the characteristics of the Korean construction industry.

To develop the proposed framework, this study undertook a comprehensive literature review of the cost items of accident loss in the construction industry and in all other industries. It was found that most of the cost items were quantified from a fragmentary perspective rather than a comprehensive perspective. In addition, it was judged that it is difficult to apply conditions based on one country, as the reliability of the supporting data and regulation become insufficient and different. Most of all, the characteristics of the construction industry were not considered. Therefore, this study organized 14 intermediate groups of cost items from earlier studies. These cost groups were then revised and added through focus group interviews and a preliminary survey to reflect the institutional aspects and the characteristics of the construction industry. Finally, the proposed framework was developed with 17 cost items. The 17 cost items were finally derived as follows: the productivity loss cost (income loss cost, tax, health insurance, national insurance, employment insurance, human cost), the compensation cost (medical care benefits, temporary disability compensation benefit, survivor’s benefits, funeral service expense, workers’ compensation insurance, settlement cost, others), and the business and administrative loss cost (penalty, fine, delay reimbursement cost, administrative loss cost).

After developing the framework, a case study was conducted on one of the nation’s largest construction companies and the obtained data were analyzed. As a result, the total fatality loss was estimated to be 2,198,260 USD/person. The subtotals of the productivity loss cost, compensation cost, and business & administrative loss cost were calculated as 1,588,842 USD/person, 298,735 USD/person, and 310,683 USD/person, respectively.

The contributions of this study are as follows. First, in terms of the research aspect, the proposed framework can be utilized to calculate a quantitative fatality loss value as opposed to the metrics used in previous research. Second, in terms of the economic aspect, the proposed framework can estimate the fatality loss considering the characteristics of the Korean construction industry. Therefore, before planning a construction project, decision makers can plan the safety investment properly to reduce the fatality loss. Third, in terms of policy aspect, when a policymaker enacts the penalty regulation that applies to the construction company considering the frequency of fatal accident and fatality loss, the results of this study can be utilized.

The limitations of this study are as follows. First, there was no previous research that quantified the human cost incurred by accidents involving fatal and injury accidents in the construction industry. In this study, the human cost related to the decline in the quality of life was used with information provided by KOTI. For this reason, when such accidents occur in the construction industry, it is difficult to calculate the human cost accurately. Second, the proposed framework was developed to calculate the fatality loss in the construction industry. However, the fatality loss could not be suggested by considering the building type and work type through the proposed framework.

Future research can present a quantitative calculation method to calculate the human cost incurred by involving fatal and injury accidents in the construction industry. Additionally, a framework can be presented to calculate the fatality loss by considering building types and work types.

## Figures and Tables

**Table 1:** Results of the literature review.

Number	Authors	Industrial Type	Cost Items	Results
1	Rikhardsson and Impgaard, 2004 [27]	Service industry Construction industry Production industry	Time Materials and components external services other costs	Two-thirds of accident costs are visible and the other one-third is non-visible. The average accident loss cost per company is USD 10,300.
2	Lebeau and Boucher, 2014 [33]	All industry	Medical cost Funeral cost Salary cost Productivity losses Administrative cost Human cost	Among the cost of occupational injuries and diseases in industry in Quebec, the cost borne by the employer is USD 11,096,083,184. Among the cost of occupational injuries and diseases in the Quebec industry, the cost borne by workers is USD 3,192,580,825. Among the cost of occupational injuries and diseases in the Quebec industry, the cost borne by the community is USD 332,268,622. The cost of occupational injuries and diseases in theconstruction industry in Quebec is USD 154,415,233.
3	Leigh et al., 2004 [34]	All industry	Medical care Lost productivity Pain and suffering	The taxicab industry has the highest average accident loss cost per worker at USD 11,528 in the United States.
4	Shalini, 2009 [37]	All industries	Lost income Productivity lost Cost of the medical treatment Cost of investigation into accident	Total lost income for all industries is 21,934,840 Rs (295,597 USD). Total lost income for construction industry is 4,108,893 Rs (55,372 USD). Total productivity lost for all industries is 766,202,274 Rs (10,325,441 USD). Total productivity lost for the construction industry is 58,857,923 Rs (793,177 USD).
5	Xiang et al., 2012 [38]	Construction industry	Medical costs	The average medical cost per injury to a worker from the United States is 2357 USD. The average medical cost per injury to a non-U.S. worker is 2351 USD.
6	Aliison et al., 2019 [39]	Construction industry	Production disturbance costs Human capital costs Medical costs Administrative costs Transfer costs Other costs	The average cost of construction accidents for a short absence is 2040 AUD (1511 USD). The average cost of construction accidents for a long absence is 22,481 AUD (16,648 USD). The average cost of construction accidents in cases involving partial incapacity is 1,400,579 AUD (1,037,171 USD). The average cost of construction accidents for a fatality is 4,377,761 AUD (3,241,863 USD).
7	Feng and Wu, 2015 [41]	Construction industry	Insured costs Medical leave wages Medical expenses Lost to productivity Cost of supervisory or staff effort Damaged equipment Consumption of first-aid materials Additional work required Fines and legal expenses, etc.	The average direct cost accounts for 0.165% of the contract sum. The average indirect cost accounts for 0.086% of the contract sum. The total accident cost accounts for 0.25% of the contract sum. The ratio of average indirect accident costs to average direct accident costs is 1:1.92.
8	Gholizadeh and Esmaeili, 2020 [43]	Construction industry	Lost time compensation cost	The cost of injuries by falls to a lower level is USD 47.89. The cost of injuries caused by being struck is USD 41.55. The cost of injuries by electricity is USD 61.49. The cost of injuries involving crushing is USD 68.67.
9	Schoonover et al., 2010 [47]	Construction industry	Compensation cost	The construction industry’s prevention index (PI) is 7, which ranks 15th out of all industries. The construction industry’s injury severity is more than twice that of other industries. The category entitled ‘Foundation, structure and building exterior contractors’ shows the highest value in construction in terms of both the PI and severity.
10	Waehrer et al., 2007 [50]	Construction industry	Hospital payments Rehabilitation Nursing home care Medical equipment Insurance administrative cost for medical claims Productivity losses Household production losses Quality of life costs, etc.	The total cost of fatalities in construction is USD 4,449,000,000. The total costs of fatal and nonfatal occupational injuries and illness in construction is USD 11,527,000,000.

Note: The exchange rate (INR/USD) is 74.11 Rs to one U.S. dollar (as of 5 August 2021). The exchange rate (AUD/USD) is 1.35 AUD to one U.S. dollar (as of 5 August 2021).

**Table 2:** Information for survey based on the proposed framework.

Tier	Required Information		Unit	Input Data	Purpose
Company information	Company name		-	Nominal scale	Company identification
	Revenue of company		Mil. USD	Ratio scale	Refer to accident loss rate
	Project name		-	Nominal scale	Project identification
	Type of building		-	Nominal scale	Project identification
	Construction cost		USD	Ratio scale	Project identification
	Construction period		Days	Interval scale	Project identification
	Gender		-	Nominal scale	Worker identification
	Age		Year	Interval scale	Refer to labor loss cost
	Career		Years	Interval scale	Worker identification
Accident information	Accident date		-	Nominal scale	Refer to delay reimbursement cost
	Accident construction type		-	Nominal scale	Refer to delay reimbursement cost
	Construction progress rate		%	Ratio value	Refer to delay reimbursement cost
	Accident work construction cost		USD	Ratio scale	Refer to delay reimbursement cost
	Accident location		-	Nominal scale	Accident identification
	Original cause materials		-	Nominal scale	Accident identification
	Assailing materials		-	Nominal scale	Accident identification
	Cause of accident		-	Nominal scale	Accident identification
Raw data on accident	Industrial accident insurance	Medical care benefits	USD	Ratio scale	Refer to compensation cost
		Temporary disability Compensation benefits	USD	Ratio scale	Refer to compensation cost
		Survivor benefits	USD	Ratio scale	Refer to compensation cost
		Funeral service expense	USD	Ratio scale	Refer to compensation cost
	Workers’ compensation insurance		USD	Ratio scale	Refer to compensation cost
	Settlement costs		USD	Ratio scale	Refer to compensation cost
	Other costs		USD	Ratio scale	Refer to compensation cost
	Penalty		USD	Ratio scale	Refer to business & administrative loss cost
	Fine		USD	Ratio scale	Refer to business & administrative loss cost
	Number of days to stop working		Days	Interval scale	Refer to business & administrative loss cost
	Type of accident work		-	Nominal scale	Refer to business & administrative loss cost
	Cost of rush work		USD	Ratio scale	Refer to business & administrative loss cost
	Accident investigation	Number of participants	Person	Nominal scale	Refer to business & administrative loss cost
		Number of days	Days	Interval scale	Refer to business & administrative loss cost

**Table 3:** Analysis methods of cost items on fatality loss.

Cost Items	Type	Proposed Calculation Method
Income loss cost	A ^a^	$\overset{n}{\underset{i=1}{\sum }}\left(W\mathrm{orker}\;\mathrm{wage}\times \frac{{\left(1+Wage\;increase\;rate\right)}^{i}}{{\left(1+Real\;discount\;rate\right)}^{i}}\right)$
Tax	A	$\overset{n}{\underset{i=1}{\sum }}\left(Worker\;income\times Tax\;rate-Progressive\;tax\right)$
Heath insurance	A	$\overset{n}{\underset{i=1}{\sum }}\left(Worker\;income\times Health\;insurance\;premium\;rate\right)$
National pension	A	$\overset{n}{\underset{i=1}{\sum }}\left(Worker\;income\times National\;pension\;premium\;rate\right)$
Employment insurance	A	$\overset{n}{\underset{i=1}{\sum }}\left(Worker\;income\times Emloyment\;insurance\;premium\;rate\right)$
Human cost	C ^c^	Human cost calculated by the Korea Transport Institute
Medical care benefits	B ^b^	‘Medical care benefits’ item on the survey
Temporary disability Compensation benefits	B	‘Temporary disability compensation benefits’ item on the survey
Survivor’s benefits	B	‘Survivor’s benefits’ item on the survey
Funeral service expense	B	‘Funeral service expense’ item on the survey
Workers’ compensation insurance	B	‘Workers’ compensation insurance’ item in survey
Settlement cost	B	‘Settlement cost’ item on the survey
Others	B	‘Other costs’ item on the survey
Penalty	B	‘Penalty’ item on the survey
Fine	B	‘Fine’ item in survey
Delay reimbursement cost	A	(Accident work construction cost of stop working period) ×(0.3 ^d^ × 0.875 ^e^ + 0.7)
Administrative loss cost	A	(Number of participant)×(Number of days)×(Worker wage)

Note: ^a^ Cost items obtained through the survey directly. ^b^ Cost items calculated using national statistical data. ^c^ Cost items calculated by the proposed method using the raw data from the survey. ^d^ The ratio of labor costs for construction works according to the Korean government. ^e^ The sum of the wage and the manpower premium according to Korean standardized specifications.

**Table 4:** Intermediate groups for cost cases extracted from previous studies.

**Group**	**Income Loss** **Cost**	**Human** **Cost**	**Medical** **Cost**	**Medical** **Leave Wages**	**Survivor’s** **Benefits**	**Funeral** **Cost**	**Compensation** **Cost**
Cases	Lost earnings Normal working time Wage cost Long term disability Injured worker’s productivity losses Lost income Salary cost Sick leaves	Holidays Quality of life value	Medical administration Rehabilitation Medical care costs Long term disability Medical insurance Medical charges Physician and allied health services Nursing & home care Payments for mental health treatment Insurance premium rise Costs of first-aid Pharmaceutical and transfer costs	Medical leave wages	Lump sum compensation for permanent incapacity or death	Burial costs Funeral service expenses	Workers’ compensations Settlement cost Compensation Employer financed fringe benefits
**Group**	**Time & Productivity** **Loss**	**Material** **Loss**	**Financial** **Loss**	**Intervention** **Cost**	**Penalty**	**Fine**	**Administrative Loss** **Cost**
Cases	(Over) Time Temporary replacements Productive hours Operational personnel time Reduced productivity Recruitment Capacity loss Reputation and morale Workplace training cost Time table schedule CEO time Cost of supervisory or staff effort Additional work Human capital costs	Materials and components Material substitution Material recovery Property damages Damage of products, equipment and machinery Property insurance	Indemnity administration Fringe benefits Re-staffing Disruption Investments Vendors, consultants and contract labor expenses Property damage insurance Reduction in waste and energy use Overhead Added marginal cost Loss of market share and output Cleanup and repair	Consultants and legal support Intervention cost Legal fees Legal expenses Cost of judicial proceedings Costs arising from possible labor disputes	Penalties	Fines	Police, fire, emergency transport Coroner services Administrative cost Investigation costs Lost productivity due to investigations or inspections for accidents Administrative liability Social Security liability Contractual civil liability

**Table 5:** Results of criteria selection for accident loss by reviewing previous studies.

**Author**	**Income Loss** **Cost**	**Human** **Cost**	**Medical** **Cost**	**Medical** **Leave Wages**	**Survivor’s** **Benefits**	**Funeral** **Cost**	**Compensation** **Cost**
Rikhardsson and Impgaard, 2004 [27]	●		●				
Linhard, 2005 [28]			●				●
Gavious et al., 2009 [31]			●				
Lebeau et al., 2014 [33]	●	●	●			●	●
Shalini, 2009 [37]	●		●				
Allison et al., 2019 [39]			●				
Cressler et al., 2016 [40]			●				
Feng, 2015 [41]	●		●	●	●		
Ibarrondo-Dávila et al., 2015 [44]			●				
Shohet et al., 2018 [48]			●				
Leigh et al., 2006 [49]	●	●	●			●	
Leigh et al., 2000 [57]	●		●				
Bergstrom, 2005 [58]	●	●					
Lahiri et al., 2005 [59]			●				
Oxenburgh and Marlow, 2005 [60]	●		●				
Hinze et al., 2006 [61]			●				
Paez et al., 2006 [62]			●				
**Author**	**Time & Productivity** **loss**	**Material** **loss**	**Financial** **loss**	**Intervention** **cost**	**Penalty**	**Fine**	**Administrative loss** **cost**
Rikhardsson and Impgaard, 2004 [27]	●	●		●		●	
Linhard, 2005 [28]	●	●	●	●	●	●	
Gavious et al., 2009 [31]	●	●	●			●	●
Lebeau et al., 2014 [33]	●						●
Shalini, 2009 [37]	●						
Allison et al., 2019 [39]			●				●
Cressler et al., 2016 [40]	●						
Feng, 2015 [41]	●	●	●	●	●		●
Ibarrondo-Dávila et al., 2015 [44]	●	●	●	●	●		●
Shohet et al., 2018 [48]	●	●	●				●
Leigh et al., 2006 [49]	●	●					●
Leigh et al., 2000 [57]			●				
Bergstrom, 2005 [58]	●		●				
Lahiri et al., 2005 [59]				●			
Oxenburgh and Marlow, 2005 [60]	●		●	●	●		
Paez et al., 2006 [62]	●						●

**Table 6:** Results of framework development on fatality loss.

Cost Items				Concept and Details
Productivity loss cost	Labor loss cost	Income loss cost		• Loss of income for retirement (age of 65) due to a worker’s death
		Tax		• Loss on taxes payable until retirement incurred due to a worker’s death
		Social insurance	Heath insurance	• Loss of health insurance payable until retirement incurred due to the death of a worker
			National pension	• Loss expenses for the national pension that can be paid until retirement incurred due to the death of a worker
			Employment insurance	• Loss of employment insurance payable until retirement incurred due to the death of a worker
	Human cost			• Mental pain, sadness, or pressure felt by the bereaved family members of a worker who died in an accident are converted into the cost.
Compensation cost	Industrial accident insurance	Medical care benefits		• Insurance benefits that are paid until a worker is healed when a worker is injured or sick and needs more than four days of medical care
		Temporary disability compensation benefits		• Insurance benefits paid for the period when industrial workers involved in an accident are unable to work due to medical treatment
		Survivor benefits		• Insurance benefits paid to compensate the dependents for their losses due to the death of a worker and to ensure the livelihood of the survivors
		Funeral service expense		• Insurance benefit that pays expenses for funeral services when a worker dies from a work-related accident
	Workers’ compensation insurance			• Insurance costs paid by the employer under civil law when the employee’s accident compensation amount exceeds the Labor Standards Act
	Settlement cost			• Expenses that the business owner incurs to deal with the accident related to the victim or survivors
	Others			• The sum of the cost of the time losses and material losses other than by the injured person
Business & administrative loss cost	Business loss cost	Legal fees	Penalty	• Punishment costs imposed on someone for doing something against a law or rule
			Fine	• Punishment costs in which a person is ordered to pay a sum of money because they have done something illegal or broken a rule
		Delay reimbursement cost		• Additional cost to supplement work interrupted by an accident
	Administrative loss cost			• Time cost for an industrial accident investigation by the employer and the government due to the occurrence of an accident

**Table 7:** Results of a case study on fatality loss.

Cost Items				Fatality Loss (USD/Person)			
				Case 1	Case 2	Case 3	Average
Productivity loss cost	Labor loss cost	Income loss cost		1,162,085	1,847,111	212,905	1,074,034
		Tax		181,153	329,259	26,241	178,884
		Social insurance	Heath insurance	36,489	57,999	6685	33,725
			National pension	52,294	83,120	9581	48,332
			Employment insurance	7554	12,006	1384	6981
	Human cost			246,887	246,887	246,887	246,887
Compensation cost	Industrial accident insurance	Medical care benefits		0	1063	0	354
		Temporary disability compensation benefits		0	0	0	0
		Survivor benefits		103,806	125,089	35,264	88,053
		Funeral service expense		9582	5633	13,243	9486
	Workers’ compensation insurance			43,937	0	0	14,646
	Settlement cost			43,937	237,260	206,504	162,567
	Others			0	70,886	0	23,629
Business & administrative loss cost	Business loss cost	Legal fees	Penalty	6591	7909	2197	5565
			Fine	2021	2636	0	1552
		Delay reimbursement cost		299,170	547,275	22,998	289,814
	Administrative loss cost			9525	12,179	19,551	13,752
Total cost				2,205,031	3,586,313	803,440	2,198,261

Note: The exchange rate (KRW/USD) is 1138 won to one U.S. dollar (as of 11 March 2021).

## Data Availability

The data presented in this study are available in the manuscript.
